# Traditional Japanese Herbal Medicine Hochu-Ekki-to Promotes Pneumococcal Colonization Clearance via Macrophage Activation and Interleukin 17A Production in Mice

**DOI:** 10.3389/fcimb.2020.569158

**Published:** 2020-10-22

**Authors:** Sho Nakakubo, Soichiro Kimura, Kazuyuki Mimura, Chiaki Kajiwara, Yoshikazu Ishii, Satoshi Konno, Kazuhiro Tateda

**Affiliations:** ^1^Department of Microbiology and Infectious Diseases, Faculty of Medicine, Toho University Graduate School of Medicine, Tokyo, Japan; ^2^Department of Respiratory Medicine, Faculty of Medicine and Graduate School of Medicine, Hokkaido University, Hokkaido, Japan

**Keywords:** traditional herbal medicine, Hochu-ekki-to (TJ-41), pneumococcal colonization, macrophage, interleukin 17, innate immunity

## Abstract

*Streptococcus pneumoniae* may colonize the nasopharynx, and as pneumococcal colonization causes invasive diseases and the subsequent transmission, reducing bacterial burden in the nasal cavity is critical. Hochu-ekki-to (TJ-41) is a traditional Japanese herbal medicine that exerts immunomodulatory effects in host cells. In this study, we investigated the potency of TJ-41 in modulating pneumococcal colonization clearance by activating host immunity. Mice, intranasally inoculated with pneumococci, were treated orally with TJ-41. During colonization, TJ-41 treatment significantly reduced pneumococcal burden and increased macrophage population in the nasopharynx. Furthermore, interleukin 17A production was significantly enhanced after TJ-41 treatment. *In vitro* experiment using nasal-derived cells revealed that pneumococcal antigen exposure upregulated the transcription of interleukin 17A in the TJ-41-treated group compared with that in the control group. Macrophages activated by killed bacteria were significantly increased in the presence of TJ-41 in an interleukin 17A-dependent manner. Moreover, TJ-41 enhanced phagocytosis, inhibited bacterial growth, and improved the antigen-presenting capacity of macrophages. Our results demonstrate that TJ-41 accelerates the clearance of pneumococcal nasopharyngeal colonization via macrophage activation. Subsequent production of interleukin 17A provides an additional benefit to effector cells.

## Introduction

*Streptococcus pneumoniae*, a gram-positive coccus, is a major cause of pneumonia, meningitis, and sepsis. Despite the antibiotic era, pneumococcal diseases impose a heavy burden on global health with high morbidity and mortality rates (Weiser et al., 2018). Pneumococcal vaccines have preventive effects against pneumococcal diseases (Pilishvili et al., 2010; Bonten et al., 2015; Suga et al., 2015). However, it has been reported that diseases caused by vaccine-uncovered serotypes account for a higher portion of the total pneumococcal diseases, termed “serotype replacement” (Balsells et al., 2017). Hence, there is a need for preventive strategies for pneumococcal infections.

Pneumococci generally invade human nasopharynx and establish colonization. This is a prerequisite for invasive diseases (Bogaert et al., 2004). Asymptomatically colonized individuals may act as reservoirs for inter-individual transmission of pneumococci (Kadioglu et al., 2008; Wolter et al., 2014), warranting the control of pneumococcal nasopharyngeal colonization.

Clearance of pneumococcal cells from the nasal cavity occurs through various mechanisms. An innate immune response is initiated after the recognition of pneumococci by a sensor called pattern recognizing receptor, which has an integral role in the subsequent acquired immunity (van Rossum et al., 2005; Zhang et al., 2009). Bacterial phagocytosis caused by effector cells, especially macrophages, is followed by the processing of pneumococcal cells (Davis et al., 2011). The functions of macrophages are necessary for pneumococcal clearance; and macrophage activation reduces bacterial burden (Zhang et al., 2009; Iwanaga et al., 2015). On the contrary, it has also been reported that the clearance of pneumococci cannot be achieved in the absence of T-helper 17 response and CD4-positive T cell immunity (Malley et al., 2005; Trzcinski et al., 2008; Zhang et al., 2009). These cellular responses regulate macrophage/monocyte influx in the later phase of colonization and protect against further pneumococcal infection. Thus, research on novel preventive treatments for pneumococcal diseases should focus on these dynamic interactions within the immune system.

Hochu-ekki-to (TJ-41), a traditional Japanese herbal (kampo) medicine, is clinically used to treat patients with weakened or immunocompromised conditions owing to various diseases. It exhibits immunomodulatory and protective effects against various types of viral, bacterial, and fungal infections (Abe et al., 1999; Hossain et al., 1999; Yamaoka et al., 2001; Yan et al., 2002; Dan et al., 2013). Studies have shown that innate immunity responses are enhanced by TJ-41 treatment. However, it is not clear whether TJ-41 is effective against pneumococcal colonization by inducing host defense response.

We hypothesized that TJ-41 treatment might be a novel strategy to prevent pneumococcal diseases. In the present study, we aimed to evaluate the effect of TJ-41 in a pneumococcal colonization mouse model and investigate the underlying immunological mechanisms.

## Materials and Methods

### Laboratory Animals

Specific pathogen-free, 6 to 8-week-old, female BALB/c mice were purchased from Charles River Laboratories Japan, Inc. (Yokohama, Japan). We used 6 to 10-week-old female IL-17A knockout (KO; *Il-17a*^−/−^) mice in the BALB/c background, established previously by the Institute of Medical Science, University of Tokyo (Nakae et al., 2002; Ishigame et al., 2009). All mice were maintained in the Laboratory Animal Research Centre of Toho University School of Medicine. All experiments were performed according to the guidelines for Proper Conduct of Animal Experiments (Science Council of Japan) and were approved by the Institutional Animal Care and Use Committee (approval number 18-51-386).

### Bacterial Strain and Growth Conditions

We used a clinical isolate of *Streptococcus pneumoniae* strain 741 (serotype 19F) that is stocked in the Department of Microbiology and Infectious Diseases (Toho University School of Medicine, Tokyo, Japan); this strain was also used in our previous mouse pneumonia models (Tateda et al., 1996; Yoshioka et al., 2016). *Streptococcus pneumoniae* “ATCC 6303” serotype 3 strain was obtained from the American Type Culture Collection (ATCC). The cells were incubated on Mueller–Hinton agar (Becton, Dickinson [BD] & Co., Sparks, MD, USA) supplemented with 5% defibrinated sheep blood at 37°C for 18–24 h. The culture was scraped from the agar and suspended in Todd–Hewitt broth (Difco, Detroit, MI, USA) supplemented with 0.5% yeast extract (Bacto™ Yeast Extract, BD) and cultured at 37°C in 5% CO_2_. Unlysed cells in the log phase (7 h after incubation) were collected by centrifugation. The cells were quantified by measuring the absorbance of the cell suspension at 600 nm, and then plotting the optical density on a standard curve generated using known colony-forming unit (CFU) values. The bacterial culture was then diluted to the desired concentration.

### Treatment Agent

The Kampo herbal formulation, Hochu-ekki-to extract [(TJ-41; Lot No.2170041010), provided by Tsumura Co., Tokyo], is prepared as a spray-dried powder of hot water extract composed of *Astragali radix* (4.0 g), *Atractylodis lanceae rhizoma* (4.0 g), *Ginseng radix* (4.0 g), *Angelicae radix* (3.0 g), *Bupleuri radix* (2.0 g), *Zizyphi fructus* (2.0 g), *Aurantii nobilis pericarpium* (2.0 g), *Glycyrrhizae radix* (1.5 g), *Cimicifugae rhizoma* (1.0 g), and *Zingiberis rhizoma* (0.5 g). For *in vivo* experiments, TJ-41 was dissolved in water and orally administered via gavage at a dose of 2,000 mg/kg/day, starting from 14 days before colonization. This dosage was determined based on the results of previous studies (Utsuyama et al., 2001; Yan et al., 2002). Mice in the control group were administered only water. *In vitro*, TJ-41 was mixed with Roswell Park Memorial Institute (RPMI) 1640 medium at several concentrations.

### Pneumococcal Colonization Model

BALB/c mice were anesthetized intramuscularly with ketamine 50 mg/kg body weight and xylazine 10 mg/kg, and then intranasally inoculated with *S. pneumoniae* in 10 μL of saline containing 6 × 10^4^ CFUs for asymptomatic colonization. The colonization protocol was similar to that reported previously for developing pneumococcal colonization mouse models (Mccool and Weiser, 2004; van Rossum et al., 2005; Trzcinski et al., 2008; Zhang et al., 2009). At the indicated time points, bacterial burden in the nasal wash [400 μL in phosphate-buffered saline (PBS)] was measured by plating 10-fold serial dilutions of nasal wash onto blood agar plates. The plates were subsequently incubated at 37°C under 5% CO_2_ overnight; after 24 h, CFUs were enumerated.

### Cell Analysis by Flow Cytometry

The nasal tissue and nasal-associated lymphoid tissue (NALT) were harvested from mice as described previously (Asanuma et al., 1997; Wu et al., 1997). The excised tissue was minced and incubated at 37°C under 5% CO_2_ for 50 min in Roswell Park Memorial Institute 1640 medium containing 2% fetal bovine serum, 0.5 mg/mL collagenase D (Roche, Basel, Switzerland), and 150 μg/mL DNase (Roche). The samples were passed through a 70-μm cell strainer (Falcon; Thermo Fisher Scientific, Waltham, MA, USA). The cells were centrifuged, and the red blood cells were lysed using BD Pharm Lyse (BD Biosciences). Cell suspensions with stain buffer (phosphate-buffered saline) containing 2% bovine serum albumin and 2 mM EDTA were incubated with an Fc-receptor-blocking antibody (anti-mouse CD16/32, clone 93) for 15 min on ice to reduce non-specific antibody binding. The cells were then washed with stain buffer and surface stained for 30 min on ice using each experimental design combination of peridinin chlorophyll protein complex/Cy5.5 anti-mouse CD11b antibody (clone M1/70), allophycocyanin (APC)/Cy7 anti-mouse/human CD11c antibody (clone N418), fluorescein isothiocyanate anti-mouse Ly6G antibody (clone 1A8), phycoerythrin anti-mouse CD86 (clone GL-1), phycoerythrin/Cy7 anti-mouse F4/80 antibody (clone BM8) (all from BioLegend), and APC anti-mouse MHC class II (I-A/I-E) antibody (clone M5/114.15.2; Tonbo Biosciences). Flow cytometry was performed with BD FACS-Canto II (BD Biosciences), and the results were analyzed using FlowJo software (TreeStar). The gating strategy for flow cytometry is illustrated in Supplementary Figure 1.

### Intracellular Cytokine Staining of NALT Cells

Intracytoplasmic cytokine staining of NALT cells was performed using the Cytofix/Cytoperm Plus kit according to the manufacturer's protocol (BD Biosciences), as described previously (Kusaka et al., 2018). To assess intracellular cytokine expression, the cells were treated with phosphomolybdic acid (PMA; 25 ng/mL), ionomycin (1 μg/mL), and GolgiPlug (1 μg/mL; BD Biosciences) and incubated for 4 h. The cells were then stained for cell-surface markers and fixed for 20 min on ice. After washing, the cells were stained for intracytoplasmic IL-17A expression with APC anti-mouse IL-17A (clone eBio17B7) or APC IgG isotype control diluted in Perm/Wash solution (BD Biosciences) for 30 min and detected using FACS-Canto II; the results were analyzed using FlowJo software (Supplementary Figure 1).

### Isolation of Immune Cells From Nasal Cavity and Cell Experiments *in vitro*

The nasal tissue and NALT were harvested from mice, and the cells were collected as described above. White blood cells were isolated with Percoll solution and plated at a concentration of 5 × 10^5^ cells/well in a total volume of 100 μL. The cells were stimulated with or without whole cell antigen (ethanol-killed *S. pneumoniae* serotype 19F) for 3 days with either TJ-41 (1 mg/mL) containing RPMI medium or medium only. Whole cell antigen (WCA) was prepared with ethanol as reported previously (Malley et al., 2001). Macrophage depletion was accomplished by treating the cells with clodronate liposome (30 μg/well; Hygieia Bioscience) 1 day before the experiment. Two hours after the administration of clodronate liposome, the culture medium was washed. Adherent cells were obtained by removing floating cells.

### RNA Isolation and Gene Expression Analysis

The total RNA was isolated from the tissue or cells using TRIzol Reagent (Invitrogen), according to the manufacturer's instructions. For quantitative reverse transcription polymerase chain reaction (PCR) analysis, 1 μL of total RNA was reverse transcribed using the High-Capacity cDNA Reverse Transcription Kit (Applied Biosystems, Foster City, CA). Data analysis was performed on the QuantStudio3 Real Time PCR System (Applied Biosystems) using the SYBR Green real-time RT-PCR technique. The following PCR primers were used: *IL-17A*, 5′-TTTAACTCCCTTGGCGCAAAA-3′ (forward) and 5′-CTTTCCCTCCGCATTGACAC-3′ (reverse); and β*-actin*, 5′-AGAGGGAAATCGTGCGTGAC-3′ (forward); and 5′-CAATAGTGATGACCTGGCCGT-3′ (reverse). Relative fold changes in transcript levels were calculated using the 2^−ΔΔCT^ method (where CT is the threshold cycle, 45), using the housekeeping gene that encodes β-actin as a reference standard for the amount loaded and the quality of the cDNA.

### Phagocytosis Assay

*Streptococcus pneumoniae* culture was suspended in RPMI medium with or without TJ-41. This suspension was added to macrophages pre-treated with TJ-41 for 2 h. The macrophages and bacterial cells were co-cultured for 1 h at 37°C under 5% CO_2_ in humidified air, washed twice, and treated with penicillin (20 U/mL) for 30 min. The macrophages were subsequently washed twice and lysed in distilled water. The viable counts of phagocytized *S. pneumoniae* were determined. This assay was performed using MH-S cell line, and adherent cells derived from the nasal cavity of BALB/c mice.

### Bacterial Growth Inhibition Assay

*Streptococcus pneumoniae* culture was suspended in RPMI medium and added to macrophage cell culture (MH-S). The macrophages and bacterial cells were co-cultured for 1 h at 37°C under 5% CO_2_ in humidified air, and then washed twice and treated with penicillin (20 U/mL) for 30 min. The contents in the wells were washed twice and then replaced with medium with or without TJ-41 and incubated for 2 h. Intracellular bacterial loads were measured before (0 h) and at the end of TJ-41 treatment (2 h).

### Antigen-Presenting Capacity Assay

A part of the procedure was similar to that of the bacterial growth inhibition assay. After pneumococcal uptake and incubation with or without TJ-41, macrophages (MH-S, 1 × 10^5^ cells/well) were washed and fixed with 2% paraformaldehyde (PFA) in PBS for 20 min, and then washed three times. T cells from BALB/c mouse spleen were isolated using the MACS Pan T Cell Isolation Kit (Miltenyi Biotec). The cells (1 × 10^6^ cells/well) were then added to fixed macrophages and cultured for up to 3 days under Th17-polarizing condition using mouse IL-6 (20 ng/mL; BioLegend), mouse TGF-β (1 ng/mL; BioLegend), anti-mouse IFN-γ Ab (1 μg/mL; BioLegend), anti-mouse IL-4 Ab (1 μg/mL; BioLegend), anti-mouse CD3 Ab (10 μg/mL; BioLegend), and anti-mouse CD28 Ab (5 μg/mL). IL-17A production from T cells, reflecting the degree of antigen presentation by macrophages, was measured using mouse ELISA kits (R&D Systems, MN, USA) according to the manufacturer's protocols.

### Statistical Analysis

All results are expressed as mean ± standard deviation. Data were analyzed using GraphPad Prism 8 software (GraphPad, Inc., La Jolla, CA, USA). The differences between the treatment and control groups were tested for significance using Mann–Whitney *U*-test. Statistical significance among more than three groups was determined using the one-way analysis of variance, followed by Tukey's multiple comparison *post-hoc* test for comparisons between groups. Results with *P* < 0.05 were considered statistically significant.

## Results

### Pneumococcal Colony Density Is Decreased by TJ-41 Treatment

**Figure 1:**
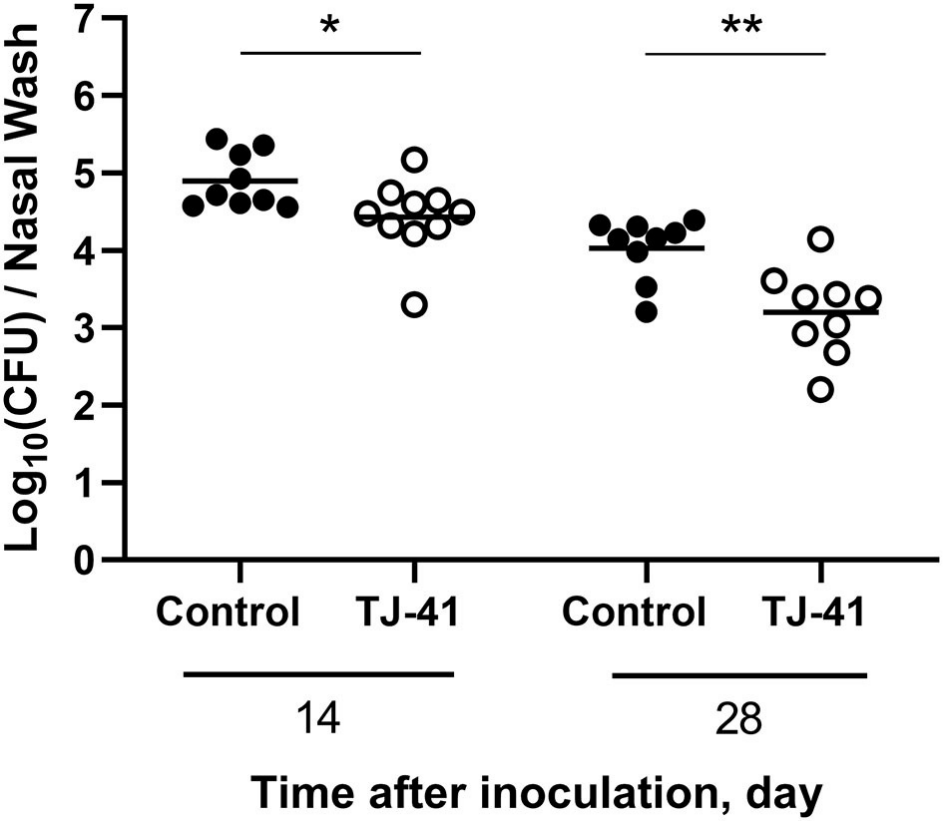
Effect of Hochu-ekki-to (TJ-41) on nasopharyngeal bacterial count in the pneumococcal colonization mouse model. This figure shows colonization density in the nasal cavity 14 and 28 days after the inoculation of pneumococcus. Each symbol represents data from one mouse, and the horizontal bars represent values for that group (*n* = 4–5 mice in each group). Black symbols and clear symbols represent the control group and TJ-41-treated group, respectively. *P < 0.05 and **P < 0.01. Data were pooled from two independent experiments. CFU, colony forming unit.

An examination of the transitional change in bacterial load in the nasal cavity of the pneumococcal colonization model revealed that it was slightly decreased, although colonization in the nasal cavity was confirmed during the period of examination (Supplementary Figure 2). As shown in Figure 1, bacterial count in the nasal wash was significantly lower in the TJ-41-treated group than in the control group on days 14 and 28 post-colonization. The results suggest that TJ-41 has a positive effect on pneumococcal colonization clearance. We confirmed that TJ-41 has no direct effect against *S. pneumoniae* (data not shown).

### TJ-41 Promotes Macrophage Activation and IL-17A Production in the Nasal Cavity

**Figure 2:**
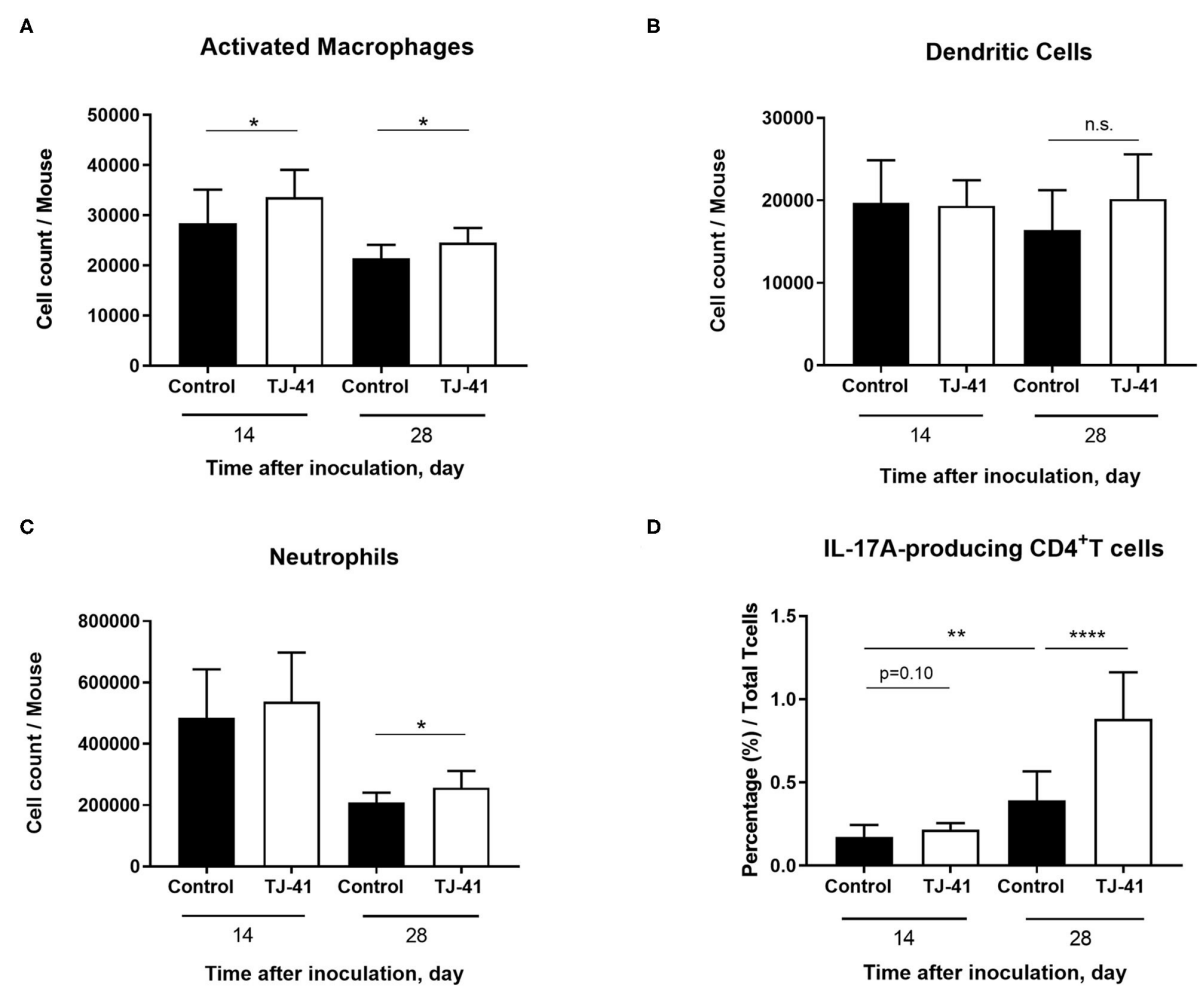
TJ-41 treatment induces changes in interleukin 17A (IL-17A) production in T cells and accumulation of macrophages in the nasal cavity during pneumococcal colonization. The number of **(A)** activated macrophages (CD86^+^F4/80^+^ cells), **(B)** dendritic cells (CD11c^+^MHC-classII^+^ cells), and **(C)** neutrophils (CD11b^+^Ly6G^+^ cells) from the nasal tissue were measured 14 and 28 days after inoculation. **(D)** Percentage of IL-17A-producing T cells in the nasal associated lymphoid tissue (NALT) was assessed 14 and 28 days after pneumococcal inoculation. The bars indicate mean ± standard deviation (*n* = 5–7 mice in each group). *P < 0.05, **P < 0.01, and ****P < 0.0001. Data were pooled from two independent experiments.

The flow cytometric analysis of the nasal tissue and NALT was performed to measure the number of inflammatory cells and the percentage of IL-17A-producing T cells, respectively. A significant increase in CD86-positive macrophages, defined as activated state, was observed in the TJ-41-treated group on days 14 and 28 (Figure 2A). There was no difference in the number of dendritic cells between the two groups (Figure 2B). On day 28, the neutrophil count in the TJ-41 treated group was significantly higher than that in the control group, but the neutrophil count in both groups was substantially lower than that on day 14 (Figure 2C). Notably, IL-17A production in TJ-41-treated mice was considerably upregulated (Figure 2D). Hence, the presence of macrophages and production of IL-17A in the nasal cavity may be key to understanding its underlying mechanisms. Furthermore, we administered TJ-41 to uncolonized mice, and examined cellular response in the nasal cavity after 6 weeks. Unlike the results observed in colonized mice, neither macrophage count nor IL-17A-producing T cell proportion differed between the uncolonized mice treated with TJ-41 and uncolonized control mice (data not shown). An assessment of the difference in anti-*S. pneumoniae* antibody titer of blood between the groups revealed no significant difference (Supplementary Figure 3).

### Effect of TJ-41 on Pneumococcal Colonization Is IL-17A Dependent

**Figure 3:**
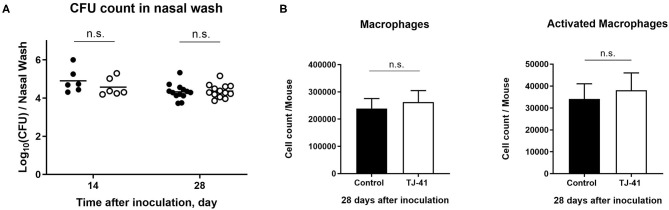
Evaluation of the effect of TJ-41 on IL-17A knockout mice (*Il-17a*^−/−^) during pneumococcal colonization. **(A)** Colonization density in the nasal cavity at 14 and 28 days after inoculation of pneumococcus. Each symbol represents data from one mouse, and the horizontal bars represent values for that group (*n* = 3 mice in each group on day 14 and *n* = 7 in each group on day 28). Black symbols and clear symbols represent the control group and TJ-41-treated group, respectively. Data were pooled from two independent experiments. **(B)** The number of macrophages (F4/80^+^ cells) and activated macrophages (CD86^+^F4/80^+^ cells) from the nasal tissues on day 28 after inoculation. The bars indicate mean ± standard deviation (*n* = 7 mice in each group). Data were pooled from two independent experiments. n.s., not significant.

To identify whether IL-17A upregulation in the TJ-41-treated group has an effect on the clearance of pneumococcal colonization, the above experiment was performed in IL-17A KO mice (Figure 3). Unlike the results in the wild-type mouse model, TJ-41 did not show a reducing effect on bacterial load in nasal wash on day 14 or 28 after pneumococcal colonization. Additionally, there was no difference in activated macrophage count between the TJ-41-treated and untreated groups. These results imply that IL-17A is necessary for enhanced pneumococcal clearance induced by TJ-41 in the nasal cavity.

### Impact of IL-17A on Macrophage Activation and Phagocytosis in TJ-41 Treatment

**Figure 4:**
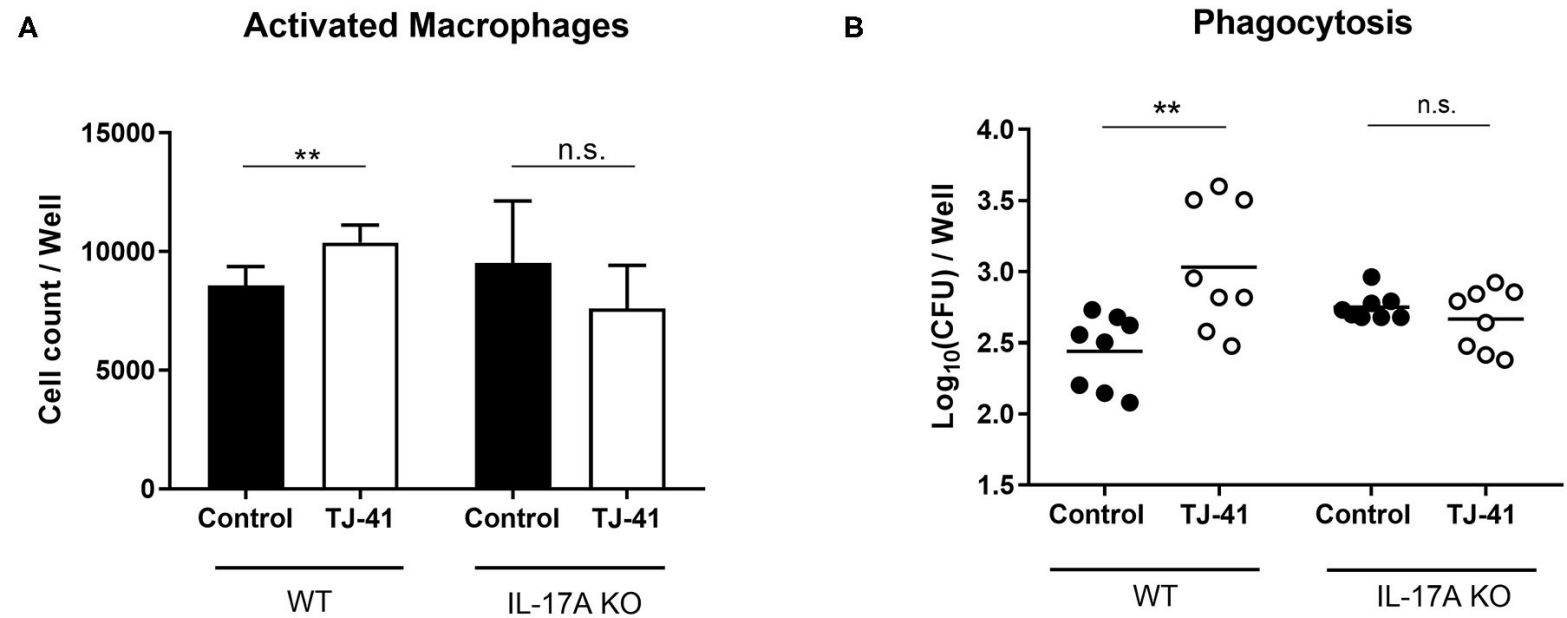
*In vitro* difference in the effect of TJ-41 on macrophages between wild type (WT) and IL-17A KO mice exposed to WCA. Cells were collected from the nasal cavity of either WT or IL-17A KO mice and incubated with WCA for 3 days. **(A)** The difference in CD86^+^F4/80^+^ macrophage count between control and TJ-41-treated cells for WT or IL-17A KO mice. The bars indicate mean ± standard deviation (*n* = 5–6 wells in each group). **(B)** Intracellular bacterial load in adherent cells after pneumococcal exposure, and this represents phagocytic activity. Adherent cells, 75% of which were macrophages, were obtained by removing floating cells. The mean number of adherent cells was 3.0 × 10^4^ cells/well and multiplicity of infection (MOI) was 33. Each symbol represents data from one well, and the horizontal bars represent values for that group. **P < 0.01, n.s., not significant. Data are representative of two independent experiments. CFU, colony forming unit.

The results presented in Figures 2, 3 raises the question whether IL-17A abundance influences macrophage activation and accumulation in the nasopharynx. Therefore, immune cells were collected from the nasal cavity *in vitro* for analysis and macrophage activation was assessed by flow cytometry. For WCA (ethanol-killed pneumococci) exposure, the TJ-41 treatment showed a significant increase in activated macrophage count in wild-type cells, but there was no significant difference in activated macrophage population between the control and TJ-41 groups of IL-17A KO cells (Figure 4A). Thereafter, we evaluated the phagocytic capacity of adherent cells; 75% of these cells were macrophages (confirmed by flow cytometry analysis), were obtained by removing floating cells (data not shown). In wild-type cells, macrophages in the TJ-41 group showed a higher uptake of pneumococcus than those in the control group (Figure 4B). No differential uptake was observed between the two groups of IL-17A KO cells (Figure 4B). The total count of macrophages did not significantly differ between the groups in both cell backgrounds (data not shown). These results suggest that TJ-41 treatment effect requires IL-17A production to provide an additional benefit of macrophage activation and phagocytosis.

### TJ-41 Stimulates IL-17A mRNA Expression of Nasopharynx-Derived Cells in a Macrophage-Dependent Manner

**Figure 5:**
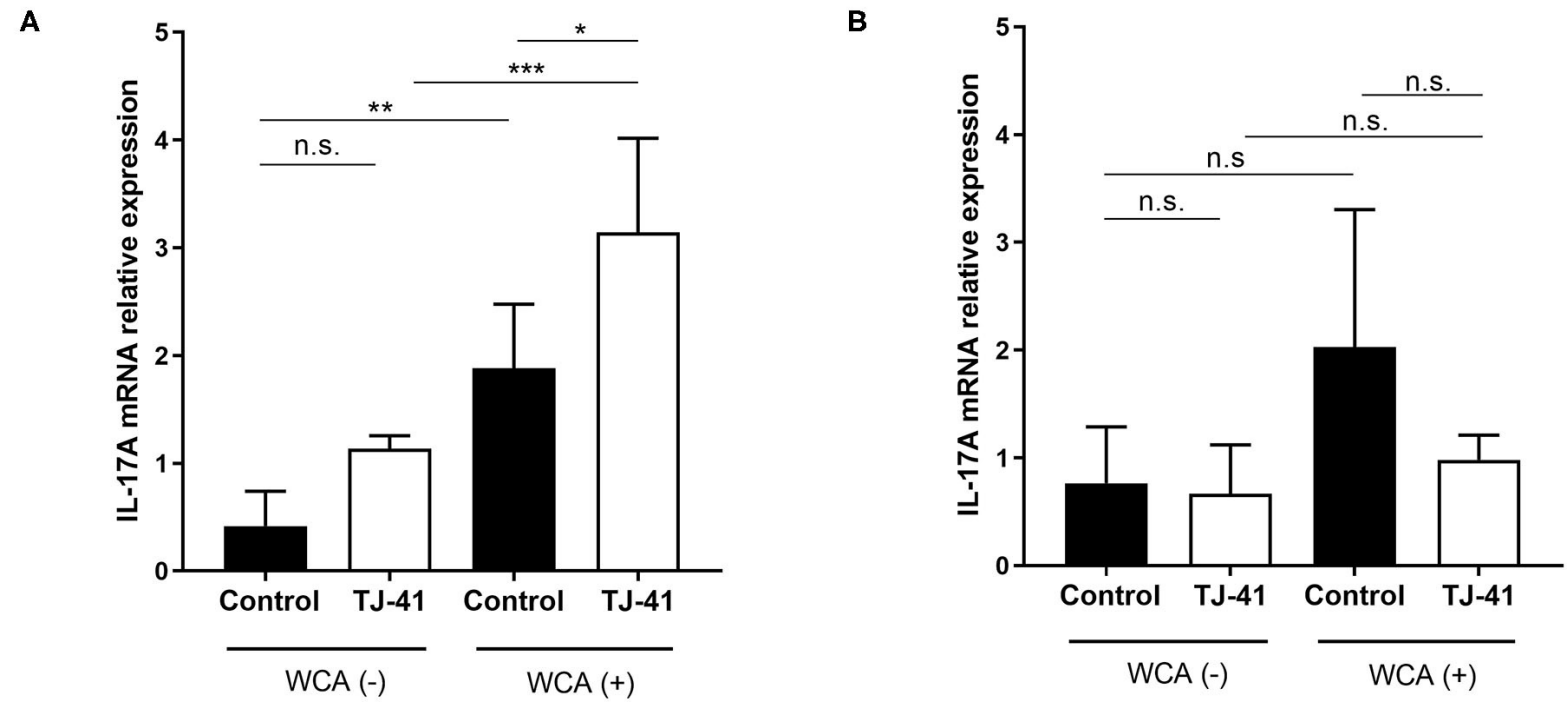
*In vitro* IL-17A messenger RNA (mRNA) expression in immune cells collected from the nasal cavity following pneumococcal antigen exposure and TJ-41 administration. Nasal cavity-derived cells were incubated with or without whole cell antigens (WCA), and each group was administered either TJ-41 or medium only. Relative expression of *IL-17A* mRNA in each group on day 3 was measured for **(A)** normal cells and **(B)** cells without macrophages; treated with clodronate-liposome on day −1. The bars indicate mean ± standard deviation (*n* = 4–7 wells in each group). *P < 0.05, **P < 0.01, and ***P < 0.001. n.s., not significant. Data are representative of three independent experiments.

To determine how TJ-41 acts on the entire immune system during pneumococcal colonization of the nasal cavity, we measured *in vitro* IL-17A mRNA expression in nasal cavity-derived immune cells. There was a significant difference in IL-17A mRNA expression between the groups with or without WCA. A comparison of groups stimulated with WCA showed a significantly higher IL-17A mRNA expression in the TJ-41 group than in the control group (Figure 5A). The same procedure was conducted by using nasal cavity-derived cells with macrophage depletion (Figure 5B). In the absence of macrophages, enhanced IL-17A expression was not observed in the TJ-41 group treated with WCA, suggesting that TJ-41 accelerates IL-17A production in the nasal cavity via macrophage regulation.

### TJ-41 Enhances Macrophage Phagocytosis of Pneumococcal Cells

**Figure 6:**
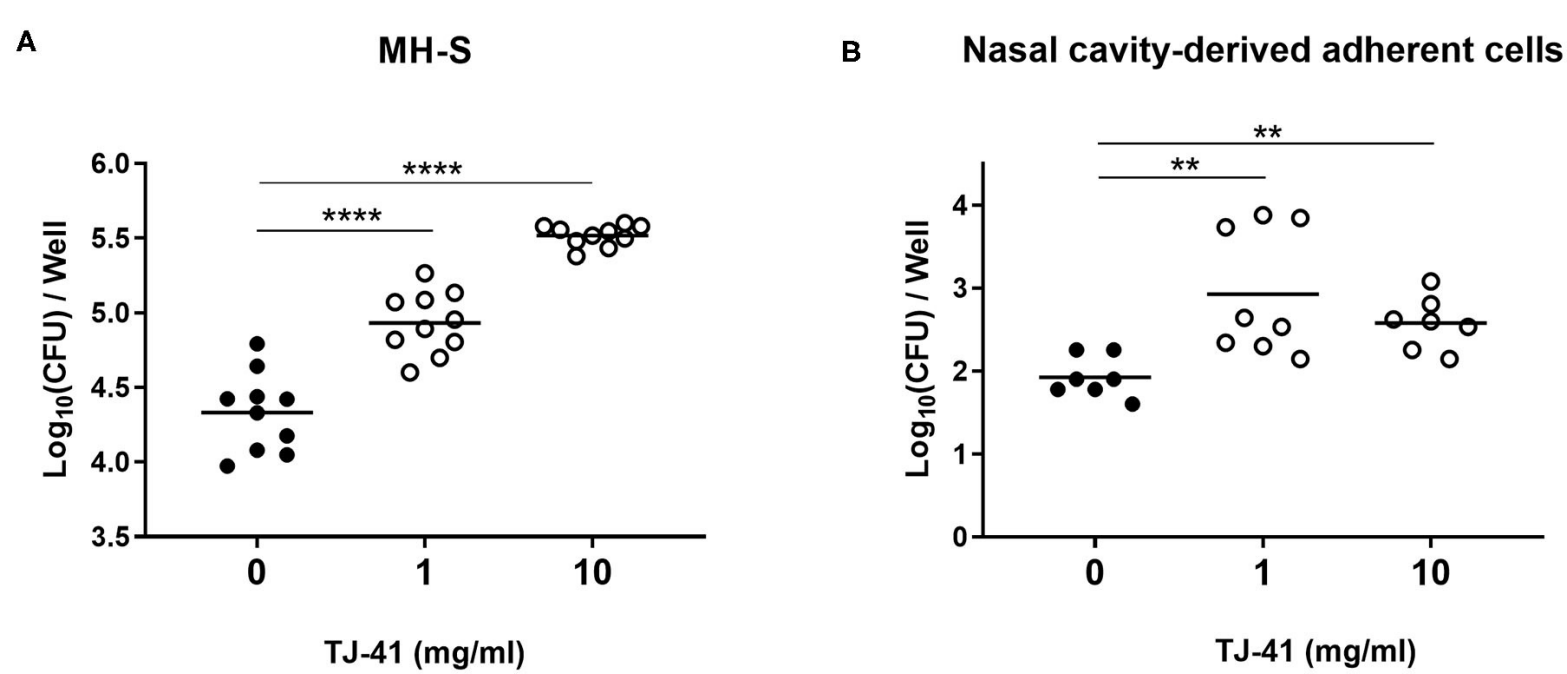
Phagocytic activity of MH-S cells or nasal cavity-derived adherent cells. **(A)** Intracellular bacterial loads of MH-S cells after pneumococcal exposure with or without TJ-41 were measured. Cells were plated at a density of 1 × 10^5^ cells/well. MOI was 10. **(B)** Phagocytic activity of adherent cells derived from the nasal cavity was measured. Mean number of adherent cells was 1.0 × 10^4^ cells/well and MOI was 100. Each symbol represents data from one well, and the horizontal bars represent values for that group. **P < 0.01 and ****P < 0.0001. CFU, colony forming unit.

We evaluated the direct immunomodulatory effects of TJ-41 on macrophages. The results of the phagocytic activity test showed considerable elevation in the intracellular bacterial burden of MH-S cells, suggesting pneumococcal uptake in a concentration-dependent manner (Figure 6A). We also confirmed that TJ-41 enhanced pneumococcal uptake in adherent cells collected from the nasal cavity (Figure 6B). Similar tendencies were observed in the phagocytic activity tests using different cell lines or by inoculation of different serotype pneumococci, suggesting that the phenomenon is not limited to a specific cell line or serotype (Supplementary Figure 4).

### Antigen-Presenting Capacity and Inhibition of Intracellular Bacterial Growth Are Enhanced by TJ-41

**Figure 7:**
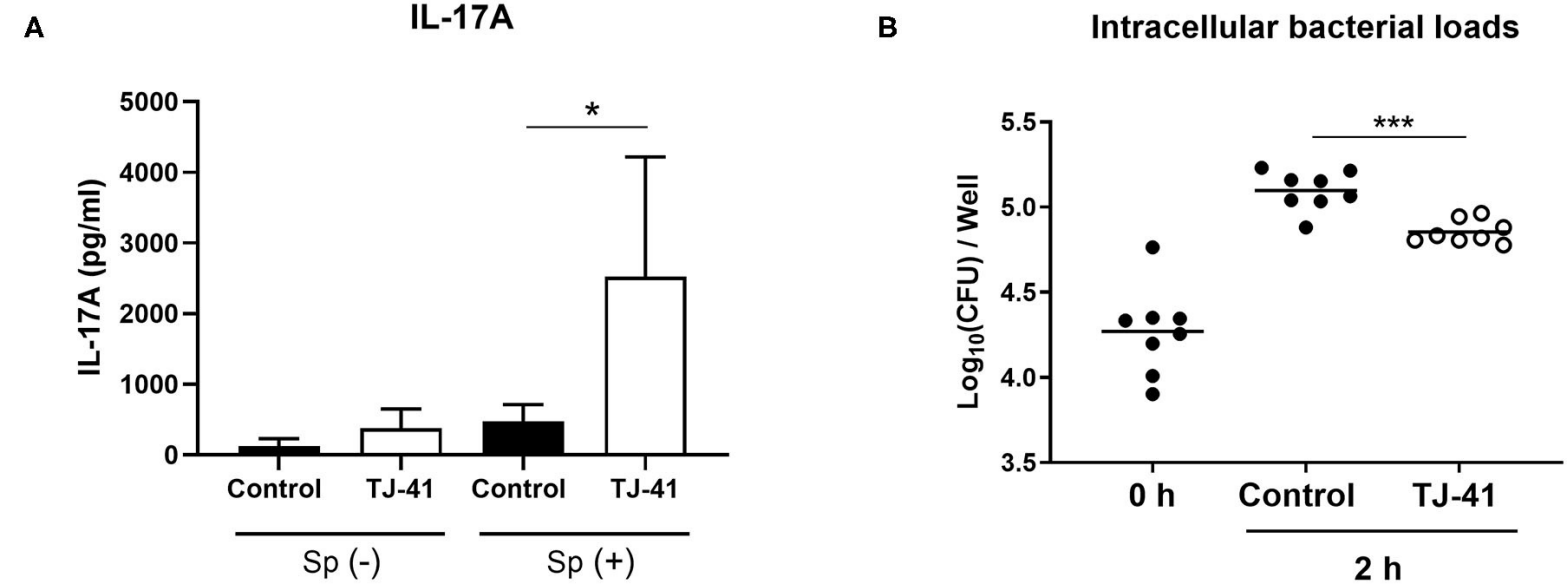
Direct effects of TJ-41 on macrophage antigen presentation to T cells and bacterial growth inhibition *in vitro*. **(A)** Assessment of the antigen-presenting capacity of MH-S cells with TJ-41 treatment. MH-S cells (1 × 10^5^ cells/well) were divided into either pneumococcal exposure (MOI = 10) or medium only group, and incubated with or without TJ-41 (1 mg/mL). Fixation using 2% PFA in PBS was performed and T cells (1 × 10^6^ cells) were added to the fixed MH-S cells under the condition of Th17 polarization. IL-17A concentration in medium on day 3 was measured using ELISA. The bars indicate mean ± standard deviation (*n* = 4 wells in each group). **(B)** Inhibitory effect of TJ-41 on bacterial growth in MH-S cells. After pneumococcal phagocytosis (MH-S: 1 × 10^5^ cells/well, MOI = 10) without any treatment, the cells were incubated for 2 h with TJ-41 (1 mg/mL) or medium only. Intracellular bacterial loads before (0 h) and after (2 h) treatment were enumerated. Each symbol represents data from one well, and the horizontal bars represent values for that group. *P < 0.05 and ***P < 0.001. These data are representative of at least two independent experiments. Sp, *Streptococcus pneumoniae*. CFU, colony forming unit.

After pneumococcal uptake and the subsequent TJ-41 treatment, MH-S cells were fixed with PFA and co-incubated with T cells to assess the antigen-presenting capacity of macrophages via cytokine production. As shown in Figure 7A, the group subjected to pneumococcal uptake and TJ-41 treatment showed a considerable increase in IL-17A production from T cells compared with that in the control group, implicating that brisk antigen presentation was induced by TJ-41. Finally, we evaluated how the bacterial count in the cells was changed by the action of TJ-41 on MH-S. After pneumococcal exposure, MH-S cells were treated with or without TJ-41. As shown in Figure 7B, the intracellular bacterial count at 2 h was higher than that at 0 h, suggesting the growth of pneumococci in MH-S cells. A comparison between the two groups at 2 h revealed that the number of bacteria in MH-S cells was significantly reduced in the TJ-41 treated group compared with that in the control group. These results indicate that TJ-41 is effective in inhibiting the growth of pneumococci in MH-S cells.

## Discussion

Here, we revealed that TJ-41 treatment promotes the clearance of pneumococcal colonization in a mouse model. Macrophage count and IL-17A production in the nasal cavity were increased in TJ-41-treated mice. Additionally, accelerated pneumococcal clearance was achieved in an IL-17A-dependent manner. Higher IL-17A abundance caused additional macrophage activation and enhanced phagocytosis. Upregulation of IL-17A mRNA expression in the nasal cavity-derived cells by TJ-41 treatment was found to be macrophage dependent. The results of MH-S cell experiments demonstrated the direct effects of TJ-41 on macrophage functions.

Nasopharyngeal colonization of *S. pneumoniae* is the first step toward the development of pneumococcal disease and transmission (Wolter et al., 2014; Weiser et al., 2018). An increase in the load of pneumococci in the nasal cavity has been shown to facilitate pneumococcal transmission in influenza A virus coinfection models (Short et al., 2012). Furthermore, Zafar et al. (2016) reported a relationship between prolonged pneumococcal shedding and high transmission rate in infant monoinfection models. Reducing bacterial burden in the nasal cavity may act as a prevention mechanism against pneumococcal infection and transmission. TJ-41 has the potential to function as a part of a preventive strategy against pneumococcal infections; however, further research is needed to confirm its efficacy in humans.

Macrophages have been shown to be major effector cells in studies on pneumococcal colonization models. Macrophage depletion allows the persistence of pneumococcal cells even in the later phase of colonization (Zhang et al., 2009). Iwanaga et al. reported that macrophage activation via macrolide treatment results in a lower burden of pneumococci in the nasopharynx (Iwanaga et al., 2015). On the basis of our results, TJ-41 modulates macrophage functions and promotes their phagocytic activity and bacterial growth inhibition. These findings suggest that the direct effects of TJ-41 on macrophages may contribute to a reduction in bacterial load in the nasal cavity to some degree. However, in accordance with the results that no significant bacterial reduction was achieved with TJ-41 treatment in the IL-17A KO mouse model, the direct activation of macrophages cannot achieve clearance by itself. Hence, in addition to macrophage functions, the effect of TJ-41 on IL-17A production should be considered when explaining the background of accelerated pneumococcal clearance.

Of note, IL-17A production in the nasal cavity is considerably upregulated in the later phase of pneumococcal colonization with TJ-41 treatment. Moreover, a similar phenomenon was observed *in vitro*; immune cells derived from the nasal cavity expressed a higher level of *IL-17A* mRNA in the presence of WCA and TJ-41, and this was found to be macrophage dependent. Furthermore, we demonstrated that macrophages exhibited a substantial increase in the antigen-presentation capacity with pneumococcal exposure and TJ-41 treatment, as evidenced by the subsequent IL-17A production from contacted T cells. Overall, it can be presumed that TJ-41 causes IL-17A upregulation via macrophage activation, especially by promoting antigen presentation. The interaction between innate immunity and IL-17A-mediated immune response is indispensable in the clearance of pneumococcal colonization (van Rossum et al., 2005; Zhang et al., 2009; Davis et al., 2011; Dorrington et al., 2013). However, whether extrinsic activation of the innate immunity provides an additional benefit of acquired immunity has not been validated. Using TJ-41 in the present study, we demonstrated the potential beneficial effect of activating innate immunity on IL-17A-mediated immune response in pneumococcal colonization.

We obtained interesting findings from the experiments using IL-17A KO mice. A significantly higher number of activated macrophages was observed after TJ-41 treatment both *in vivo* and *in vitro*. Contrary to the findings in wild-type model, the difference in the upregulation of macrophages was not observed in the IL-17A KO pneumococcal colonization model. Similarly, TJ-41 treatment did not show additional macrophage activation and enhanced phagocytosis in IL-17A KO mouse-derived nasal cells stimulated with WCA. TJ-41 may help macrophages to lower pneumococcal colonization density by direct modulation. However, a substantial decrease in bacterial load occurred as a result of the activity of IL-17A. A previous study reported that IL-17 acts as a recruitment and survival factor for macrophages (Sergejeva et al., 2005). The presence of IL-17A in the nasal cavity allows macrophages/monocytes to accumulate and clear the pneumococci (Zhang et al., 2009). Furthermore, Wright et al. demonstrated a significant and dose-dependent increase in pneumococcal killing by human alveolar macrophages when exposed to recombinant IL-17A (Wright et al., 2013). In light of these findings, the accelerated pneumococcal clearance of TJ-41 in this study can be considered a result of increased IL-17A production. Relatively higher abundance of IL-17A driven by TJ-41 may cause additive macrophage activation and accumulation. While previous studies validated the findings via exogenous administration of IL-17A, using our TJ-41 treatment model, we succeeded in demonstrating the role of endogenous IL-17A upregulation in the additional activation of macrophage and accelerated pneumococcal clearance.

Our study had some limitations. First, as TJ-41 is an extract of various types of plants, it was difficult to identify its most active components or their combinations. Because TJ-41 has a long-standing history and a variety of clinical applications, its effects and mechanisms can be attributed to one drug. However, further studies should be performed to determine the specific components that act on immunity against pneumococcal colonization, to develop a new treatment. Second, we did not assess whether the reduction in colonization density and IL-17A enhancement by TJ-41 resulted in host protection against invasive infection or pneumococcal transmission. Pneumococcal colonization has been shown to reduce bacterial burden in lung infection by inducing cellular and humoral immunity (Wilson et al., 2015). Enhancement of the IL-17A-mediated immune response by TJ-41 may contribute to additional host defenses against infection, and this should be addressed in future studies.

In summary, we employed a mouse model to reveal the efficacy of TJ-41 against pneumococcal colonization. As a result, we found that direct stimulation of macrophages by TJ-41 may be an inducer of the subsequent IL-17A enhancement and additional activation of effector cells, followed by accelerated clearance of pneumococci. Such findings shed light on a strategy for the prevention of pneumococcal diseases.

## Data Availability Statement

The raw data supporting the conclusions of this article will be made available by the authors, without undue reservation.

## Ethics Statement

This animal study was reviewed and approved by Toho University Animal Care and User Committee.

## Author Contributions

SN conceived the study, performed the procedure, analyzed the data, and wrote the manuscript. SKi conceived the study, advised on all experiments, analyzed the data, and wrote the manuscript. KM assisted with the experimental procedure. CK provided advice on the procedure and analyzed the data. YI, KT, and SKo supervised the whole experiment and proofread the manuscript. All authors approved the submitted paper.

## Conflict of Interest

The authors declare that the research was conducted in the absence of any commercial or financial relationships that could be construed as a potential conflict of interest.

## References

[B1] Abe S. Tansho S. Ishibashi H. Akagawa G. Komatsu Y. Yamaguchi H. (1999). Protection of immunosuppressed mice from lethal Candida infection by oral administration of a kampo medicine, hochu-ekki-to. Immunopharmacol. Immunotoxicol. 21, 331–342. 10.3109/08923979909052766 10319284

[B2] Asanuma H. Thompson A. H. Iwasaki T. Sato Y. Inaba Y. Aizawa C.. (1997). Isolation and characterization of mouse nasal-associated lymphoid tissue. J. Immunol. Methods 202, 123–131. 10.1016/S0022-1759(96)00243-8 9107301

[B3] Balsells E. Guillot L. Nair H. Kyaw M. H. (2017). Serotype distribution of *Streptococcus pneumoniae* causing invasive disease in children in the post-PCV era: a systematic review and meta-analysis. PLoS ONE 12:e0177113. 10.1371/journal.pone.0177113 28486544 PMC5423631

[B4] Bogaert D. De Groot R. Hermans P. W. (2004). *Streptococcus pneumoniae* colonisation: the key to pneumococcal disease. Lancet Infect. Dis. 4, 144–154. 10.1016/S1473-3099(04)00938-7 14998500

[B5] Bonten M. J. Huijts S. M. Bolkenbaas M. Webber C. Patterson S. Gault S.. (2015). Polysaccharide conjugate vaccine against pneumococcal pneumonia in adults. N. Engl. J. Med. 372, 1114–1125. 10.1056/NEJMoa1408544 25785969

[B6] Dan K. Akiyoshi H. Munakata K. Hasegawa H. Watanabe K. (2013). A Kampo (traditional Japanese herbal) medicine, Hochuekkito, pretreatment in mice prevented influenza virus replication accompanied with GM-CSF expression and increase in several defensin mRNA levels. Pharmacology 91, 314–321. 10.1159/000350188 23796966

[B7] Davis K. M. Nakamura S. Weiser J. N. (2011). Nod2 sensing of lysozyme-digested peptidoglycan promotes macrophage recruitment and clearance of *S. pneumoniae* colonization in mice. J. Clin. Invest. 121, 3666–3676. 10.1172/JCI57761 21841315 PMC3163965

[B8] Dorrington M. G. Roche A. M. Chauvin S. E. Tu Z. Mossman K. L. Weiser J. N.. (2013). MARCO is required for TLR2- and Nod2-mediated responses to *Streptococcus pneumoniae* and clearance of pneumococcal colonization in the murine nasopharynx. J. Immunol. 190, 250–258. 10.4049/jimmunol.1202113 23197261 PMC3529821

[B9] Hossain M. S. Takimoto H. Hamano S. Yoshida H. Ninomiya T. Minamishima Y.. (1999). Protective effects of hochu-ekki-to, a Chinese traditional herbal medicine against murine cytomegalovirus infection. Immunopharmacology 41, 169–181. 10.1016/S0162-3109(98)00066-6 10428645

[B10] Ishigame H. Kakuta S. Nagai T. Kadoki M. Nambu A. Komiyama Y.. (2009). Differential roles of interleukin-17A and−17F in host defense against mucoepithelial bacterial infection and allergic responses. Immunity 30, 108–119. 10.1016/j.immuni.2008.11.009 19144317

[B11] Iwanaga N. Nakamura S. Oshima K. Kajihara T. Takazono T. Miyazaki T.. (2015). Macrolides promote CCL2-mediated macrophage recruitment and clearance of nasopharyngeal pneumococcal colonization in mice. J. Infect. Dis. 212, 1150–1159. 10.1093/infdis/jiv157 25767216

[B12] Kadioglu A. Weiser J. N. Paton J. C. Andrew P. W. (2008). The role of Streptococcus pneumoniae virulence factors in host respiratory colonization and disease. Nat. Rev. Microbiol. 6, 288–301. 10.1038/nrmicro1871 18340341

[B13] Kusaka Y. Kajiwara C. Shimada S. Ishii Y. Miyazaki Y. Inase N.. (2018). Potential role of Gr-1+ CD8+ T lymphocytes as a source of interferon-gamma and M1/M2 polarization during the acute phase of murine legionella pneumophila pneumonia. J. Innate Immun. 10, 328–338. 10.1159/000490585 30021216 PMC6757147

[B14] Malley R. Lipsitch M. Stack A. Saladino R. Fleisher G. Pelton S.. (2001). Intranasal immunization with killed unencapsulated whole cells prevents colonization and invasive disease by capsulated pneumococci. Infect. Immun. 69, 4870–4873. 10.1128/IAI.69.8.4870-4873.2001 11447162 PMC98576

[B15] Malley R. Trzcinski K. Srivastava A. Thompson C. M. Anderson P. W. Lipsitch M. (2005). CD4+ T cells mediate antibody-independent acquired immunity to pneumococcal colonization. Proc. Natl. Acad. Sci. U. S. A. 102, 4848–4853. 10.1073/pnas.0501254102 15781870 PMC555733

[B16] Mccool T. L. Weiser J. N. (2004). Limited role of antibody in clearance of Streptococcus pneumoniae in a murine model of colonization. Infect. Immun. 72, 5807–5813. 15385481 10.1128/IAI.72.10.5807-5813.2004 PMC517579

[B17] Nakae S. Komiyama Y. Nambu A. Sudo K. Iwase M. Homma I.. (2002). Antigen-specific T cell sensitization is impaired in IL-17-deficient mice, causing suppression of allergic cellular and humoral responses. Immunity 17, 375–387. 10.1016/S1074-7613(02)00391-6 12354389

[B18] Pilishvili T. Lexau C. Farley M. M. Hadler J. Harrison L. H. Bennett N. M.. (2010). Sustained reductions in invasive pneumococcal disease in the era of conjugate vaccine. J. Infect. Dis. 201, 32–41. 10.1086/648593 19947881

[B19] Sergejeva S. Ivanov S. Lotvall J. Linden A. (2005). Interleukin-17 as a recruitment and survival factor for airway macrophages in allergic airway inflammation. Am. J. Respir. Cell Mol. Biol. 33, 248–253. 10.1165/rcmb.2004-0213OC 15901616

[B20] Short K. R. Reading P. C. Wang N. Diavatopoulos D. A. Wijburg O. L. (2012). Increased nasopharyngeal bacterial titers and local inflammation facilitate transmission of *Streptococcus pneumoniae. MBio* 3:e00255-12. 10.1128/mBio.00255-12 PMC3518912 23015738

[B21] Suga S. Chang B. Asada K. Akeda H. Nishi J. Okada K.. (2015). Nationwide population-based surveillance of invasive pneumococcal disease in Japanese children: effects of the seven-valent pneumococcal conjugate vaccine. Vaccine 33, 6054–6060. 10.1016/j.vaccine.2015.07.069 26235372

[B22] Tateda K. Takashima K. Miyazaki H. Matsumoto T. Hatori T. Yamaguchi K. (1996). Noncompromised penicillin-resistant pneumococcal pneumonia CBA/J mouse model and comparative efficacies of antibiotics in this model. Antimicrob. Agents Chemother. 40, 1520–1525. 10.1128/AAC.40.6.1520 8726030 PMC163360

[B23] Trzcinski K. Thompson C. M. Srivastava A. Basset A. Malley R. Lipsitch M. (2008). Protection against nasopharyngeal colonization by *Streptococcus pneumoniae* is mediated by antigen-specific CD4+ T cells. Infect. Immun. 76, 2678–2684. 10.1128/IAI.00141-08 18391006 PMC2423086

[B24] Utsuyama M. Seidlar H. Kitagawa M. Hirokawa K. (2001). Immunological restoration and anti-tumor effect by Japanese herbal medicine in aged mice. Mech. Ageing Dev. 122, 341–352. 10.1016/S0047-6374(00)00249-9 11311321

[B25] van Rossum A. M. Lysenko E. S. Weiser J. N. (2005). Host and bacterial factors contributing to the clearance of colonization by *Streptococcus pneumoniae* in a murine model. Infect. Immun. 73, 7718–7726. 10.1128/IAI.73.11.7718-7726.2005 16239576 PMC1273875

[B26] Weiser J. N. Ferreira D. M. Paton J. C. (2018). *Streptococcus pneumoniae*: transmission, colonization and invasion. Nat. Rev. Microbiol. 16, 355–367. 10.1038/s41579-018-0001-8 29599457 PMC5949087

[B27] Wilson R. Cohen J. M. Jose R. J. de Vogel C. Baxendale H. Brown J. S. (2015). Protection against Streptococcus pneumoniae lung infection after nasopharyngeal colonization requires both humoral and cellular immune responses. Mucosal Immunol. 8, 627–639. 10.1038/mi.2014.95 25354319 PMC4351900

[B28] Wolter N. Tempia S. Cohen C. Madhi S. A. Venter M. Moyes J.. (2014). High nasopharyngeal pneumococcal density, increased by viral coinfection, is associated with invasive pneumococcal pneumonia. J. Infect. Dis. 210, 1649–1657. 10.1093/infdis/jiu326 24907383

[B29] Wright A. K. Bangert M. Gritzfeld J. F. Ferreira D. M. Jambo K. C. Wright A. D.. (2013). Experimental human pneumococcal carriage augments IL-17A-dependent T-cell defence of the lung. PLoS Pathog 9, e1003274. 10.1371/journal.ppat.1003274 23555269 PMC3610738

[B30] Wu H. Y. Nguyen H. H. Russell M. W. (1997). Nasal lymphoid tissue (NALT) as a mucosal immune inductive site. Scand. J. Immunol 46, 506–513. 10.1046/j.1365-3083.1997.d01-159.x 9393634

[B31] Yamaoka Y. Kawakita T. Nomoto K. (2001). Protective effect of a traditional Japanese medicine Hochu-ekki-to (Chinese name: Bu-zhong-yi-qi-tang), on the susceptibility against Listeria monocytogenes in infant mice. Int. Immunopharmacol 1, 1669–1677. 10.1016/S1567-5769(01)00076-5 11562059

[B32] Yan X. Kita M. Minami M. Yamamoto T. Kuriyama H. Ohno T.. (2002). Antibacterial effect of Kampo herbal formulation Hochu-ekki-to (Bu-Zhong-Yi-Qi-Tang) on Helicobacter pylori infection in mice. Microbiol. Immunol 46, 475–482. 10.1111/j.1348-0421.2002.tb02721.x 12222933

[B33] Yoshioka D. Kajiwara C. Ishii Y. Umeki K. Hiramatsu K. Kadota J.. (2016). Efficacy of beta-lactam-plus-macrolide combination therapy in a mouse model of lethal pneumococcal pneumonia. Antimicrob. Agents Chemother 60, 6146–6154. 10.1128/AAC.01024-16 27480866 PMC5038280

[B34] Zafar M. A. Kono M. Wang Y. Zangari T. Weiser J. N. (2016). Infant mouse model for the study of shedding and transmission during *Streptococcus pneumoniae* monoinfection. Infect. Immun. 84, 2714–2722. 10.1128/IAI.00416-16 27400721 PMC4995895

[B35] Zhang Z. Clarke T. B. Weiser J. N. (2009). Cellular effectors mediating Th17-dependent clearance of pneumococcal colonization in mice. J. Clin. Invest. 119, 1899–1909. 10.1172/JCI36731 19509469 PMC2701860

