# Supplementary material for: Traditional Japanese Herbal Medicine Hochu-Ekki-to Promotes Pneumococcal Colonization Clearance via Macrophage Activation and Interleukin 17A Production in Mice
Source: Front Cell Infect Microbiol. 2020 Oct 22;10:569158. doi: 10.3389/fcimb.2020.569158 (PMC7649813; doi:10.3389/fcimb.2020.569158)
Supplement: Supplementary file 1 [file Data_Sheet_1.pdf]

Supplementary Material

1. Supplementary Figures

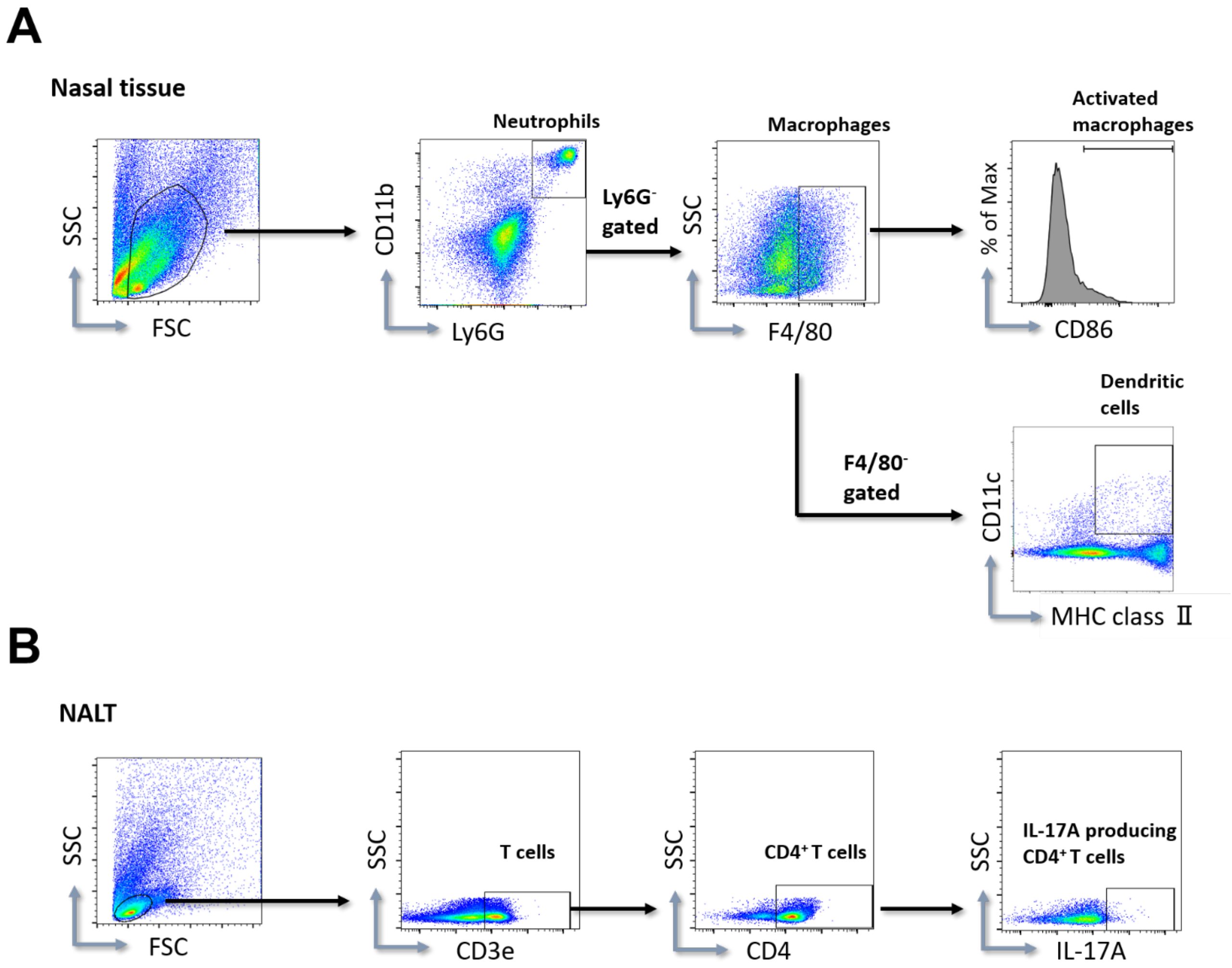

Supplementary Figure 1. Gating strategy for the flow cytometry analysis. (A) Nasal tissues were harvested and analyzed by flow cytometry. Neutrophils, macrophages, activated macrophages, and dendritic cells were defined as follows. $Ly6G^+CD11b^+$, neutrophils; $F4/80^+Ly6G^-$, macrophages; $CD86^+F4/80^+Ly6G^-$, activated macrophages; $CD11c^+$MHC class Ⅱ$^+F4/80^-Ly6G^-$, dendritic cells. (B) Nasal associated lymphoid tissue (NALT) was harvested and analyzed. IL-17A-producing $CD4^+$ T cells were defined as $CD3e^+CD4^+IL\text{-}17A^+$.

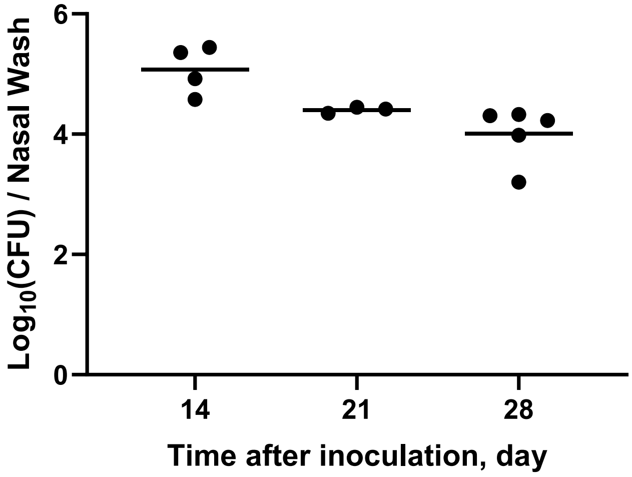

Supplementary Figure 2. Natural course of nasopharyngeal bacterial load in the pneumococcal colonization mouse model. The figure shows colonization density in the nasal cavity after inoculating pneumococcus. Each symbol represents data from one mouse, and the horizontal bars represent values for that group (n = 3–5 mice). Black symbols and clear symbols represent the control group and TJ-41-treated group, respectively. Abbreviations: CFU, colony forming unit.

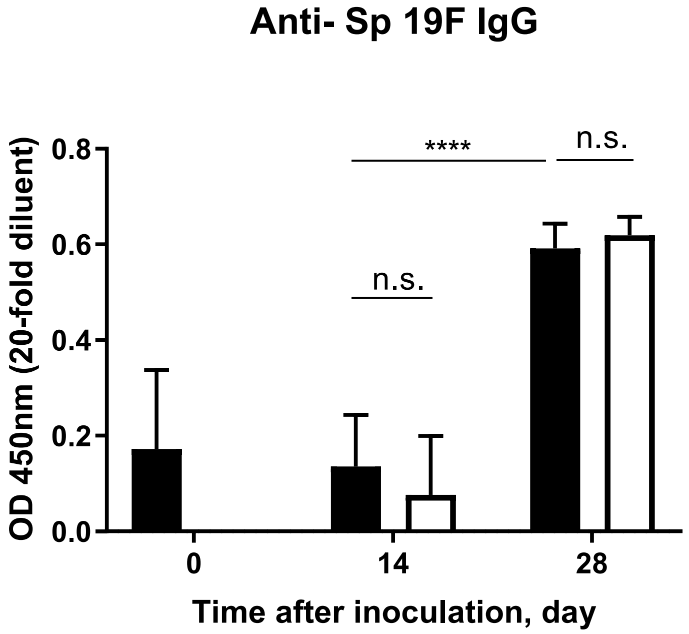

Supplementary Figure 3. Antibody response in the pneumococcal colonization mouse model. Anti-serotype 19F strain IgG titer in serum was measured on days 14 and 28 after colonization by whole-cell ELISA as described previously (Wilson et al., 2015). The black and clear bars represent the control group and TJ-41 treated group, respectively. The bars indicate mean ± standard deviation. Absorbance was read at 450 nm. Data are representative of two independent experiments (n = 3–5 mice in each group). ****P < 0.0001, n.s., not significant.

Wilson, R., Cohen, J.M., Jose, R.J., De Vogel, C., Baxendale, H., and Brown, J.S. (2015). Protection against Streptococcus pneumoniae lung infection after nasopharyngeal colonization requires both humoral and cellular immune responses. *Mucosal Immunol* 8**,** 627-639.

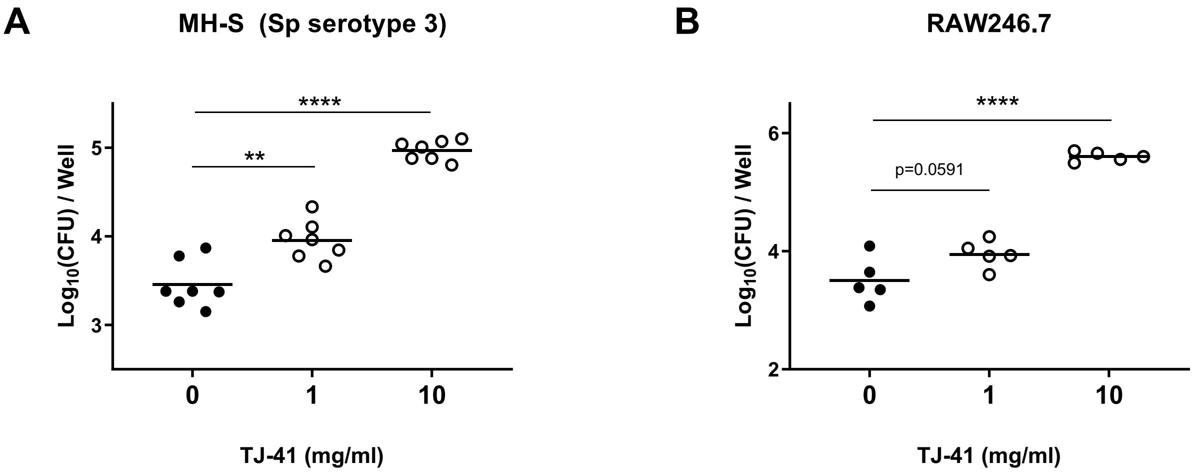

Supplementary Figure 4. Direct effects of TJ-41 on the phagocytic activity of macrophages. (A) Phagocytic activity of MH-S cells exposed to *Streptococcus pneumoniae* serotype 3 (ATCC 6303). Intracellular bacterial loads of MH-S cells (1×10^5 cells/well) after pneumococcal exposure (MOI = 10) with or without TJ-41 were measured. (B) Phagocytic activity of RAW246.7 cells. Procedure was the same as above (1×10^5 cells/well, bacteria: *Streptococcus pneumoniae* serotype 19F, MOI = 10). Each symbol represents the data from one well, and the horizontal bars indicate values for that group. $^{**}P < 0.01$, $^{****}P < 0.0001$. Abbreviation: CFU, colony forming unit.
